# Psychometric validation of the Chinese Physical Effort Scale and profiles of physical-effort approach and avoidance among Chinese university students

**DOI:** 10.3389/fpsyg.2026.1959219

**Published:** 2026-10-16

**Authors:** Yijun Liu, Qiang Zhou

**Affiliations:** School of Physical Education, Nanchang University, Nanchang, China

**Keywords:** exercise motivation, individual differences, physical activity, physical effort perception, Physical Effort Scale, psychometric validation

## Abstract

**Objective:**

Individual differences in tendencies to approach or avoid physical effort may be relevant to exercise-related psychological and behavioral characteristics. This study examined the psychometric properties of the Chinese Physical Effort Scale (PES) and investigated individual differences in physical-effort approach and avoidance tendencies and their associations with exercise-related psychological and behavioral variables among Chinese university students.

**Methods:**

Participants were 1,273 students from six universities across eastern, central, and western China. Exploratory and confirmatory factor analyses, reliability and validity analyses, and multigroup confirmatory factor analysis were used to evaluate the factor structure, reliability, validity, and measurement invariance of the Chinese PES. Latent profile analysis identified configurations of physical effort approach and avoidance, and BCH analyses compared profiles on emotion regulation, exercise motivation, and physical activity.

**Results:**

Factor analyses supported a two-factor structure comprising physical effort approach and avoidance. The scale showed high internal consistency and test–retest reliability, with evidence supporting convergent and discriminant validity and metric and scalar invariance across gender and academic year. Three profiles were identified: Balanced (59.07%), Avoidance-Oriented (14.93%), and Approach-Oriented (26.00%). The Approach-Oriented profile showed higher cognitive reappraisal, expressive suppression, external regulation, introjected regulation, and autonomous regulation, and lower amotivation. It also showed higher total physical activity than the other profiles and higher weekly exercise frequency than the Balanced profile.

**Conclusion:**

The Chinese PES showed psychometric support among Chinese university students. Distinct configurations of physical effort approach and avoidance were associated with emotion regulation, exercise motivation, and physical activity.

## 1 Introduction

Physical effort and the perception of effort are conceptually related but distinct. Physical effort refers to the resources required or mobilized to perform an action, whereas the perception of effort refers to the subjective experience of how effortful an action feels (Pageaux, 2016; Halperin and Vigotsky, 2024). Although the perception of effort may be associated with peripheral physiological signals, such as muscular and respiratory responses (de la Garanderie et al., 2023; Lopes et al., 2022), it is also related to higher-order psychological processes, including motivation, attentional allocation, and self-regulation (Hutchinson, 2021). Accordingly, subjective effort cannot be understood solely as a direct reflection of physiological demand or physical activity intensity; its experience and evaluation may also vary according to cognitive, motivational, and contextual factors (Cos, 2017; Halperin and Vigotsky, 2024). Such effort-related experiences are relevant to behavioral decisions concerning whether to initiate, sustain, or terminate physically demanding actions (Cheval et al., 2024, 2025). Research across exercise and health contexts further suggests that individual differences in effort-related experiences may be relevant to physical activity participation and adherence (Bevan et al., 2020; Collado-Mateo et al., 2021; Rodrigues et al., 2018). Although the perception of effort and tendencies to approach or avoid physical effort are distinct constructs, research on effort perception provides relevant conceptual context for understanding how individuals experience and respond to physical effort. The present study, however, focuses specifically on individual differences in tendencies to approach or avoid physical effort rather than on momentary perceptions of effort.

In exercise research, perceived effort is commonly assessed using the Rating of Perceived Exertion (RPE), which provides a practical method for monitoring the perceived intensity of effort during an ongoing bout of exercise (Borg, 1982; Grummt et al., 2024). RPE therefore primarily captures a momentary, state-like experience of effort or exertion associated with a specific task or exercise bout (Pageaux, 2016; Halperin and Emanuel, 2020). The Physical Effort Scale (PES), in contrast, was developed to assess a different aspect of individuals’ relationship with physical effort. Rather than quantifying how effortful a particular activity feels at a given moment, the PES assesses broader individual differences in tendencies to approach or avoid physical effort (Cheval et al., 2024). Thus, RPE and PES concern related but conceptually distinct aspects of physical effort: RPE focuses primarily on momentary perceived effort, whereas the PES focuses on broader approach and avoidance tendencies toward physical effort. Variations in RPE administration and rating procedures may also affect comparability across studies (Torres et al., 2025). More broadly, research on perceived effort has shown that effort-related experiences are associated not only with physiological demands but also with motivational, affective, and cognitive processes (Herzberg et al., 2026; Pageaux and Lepers, 2018; Smirmaul, 2012). However, conventional measures of perceived effort do not typically assess broader individual differences in approach and avoidance tendencies toward physical effort (Cheval et al., 2024, 2025; Halperin and Emanuel, 2020; Pageaux, 2016). Evidence regarding perceived exertion has also been obtained across different populations and settings, underscoring the importance of examining the measurement properties of effort-related constructs when they are applied in new populations and cultural contexts (Chen et al., 2002; Halperin and Emanuel, 2020).

To assess these broader individual differences, the Physical Effort Scale (PES) was developed to measure tendencies to approach and avoid physical effort from a behavioral-tendency perspective (Cheval et al., 2024). The PES comprises two dimensions–physical-effort approach and physical-effort avoidance–which are conceptualized as related but distinguishable tendencies rather than as opposite poles of a single bipolar construct. Accordingly, individuals may vary on each dimension, and their standing on one dimension does not conceptually determine their standing on the other. Previous research has provided initial evidence for the psychometric properties of the PES and has shown associations between its dimensions and physical activity-related variables (Cheval et al., 2024). The PES has also been adapted and psychometrically evaluated in another cultural and linguistic context, including a Japanese version (Soga et al., 2026). However, previous studies have primarily focused on scale development and psychometric evaluation. Less is known about how physical-effort approach and avoidance jointly vary across individuals and whether different empirical configurations of these tendencies are associated with exercise-related psychological and behavioral variables. Examining their joint distribution may therefore provide a person-centered complement to analyses that consider each dimension separately.

The present study aimed to examine individual differences in approach and avoidance tendencies toward physical effort and their psychological and behavioral correlates among Chinese university students. Specifically, we first evaluated the psychometric properties of the Chinese PES, including its reliability, factor structure, and measurement invariance. Physical-effort approach and avoidance were treated as related but distinguishable dimensions. We expected approach to be positively associated with autonomous exercise regulation and physical activity and negatively associated with amotivation, with the opposite pattern expected for avoidance. Associations with other psychological variables were examined exploratorily. We then used latent profile analysis (LPA) to characterize the joint distribution of approach and avoidance and to examine whether the identified profiles differed in emotion regulation, exercise motivation, and physical activity.

## 2 Materials and methods

### 2.1 Participants

Prior to data collection, we planned to recruit approximately 1,200 participants. The target sample size was determined based on the requirements of the planned psychometric analyses, including exploratory and confirmatory factor analyses, rather than a formal *a priori* power analysis (MacCallum et al., 1999; Wolf et al., 2013). A total of 1,358 questionnaires were collected, and 85 were excluded due to incomplete or invalid responses, resulting in a final sample of 1,273 participants.

Participants were recruited from six universities in eastern, central, and western China (Southwest University, Ningbo University, Henan University, Nanchang University, Jiangxi Normal University, and Nanchang Hangkong University). Eligible participants were current university students aged 18 years or older; no upper age limit was imposed. Participants were also required to be able to complete the Chinese questionnaire independently. Because the study examined exercise-related psychological variables and habitual physical activity, participants were excluded if they self-reported a physical condition that substantially restricted their usual participation in physical activity. Participants who self-reported cognitive difficulties that prevented them from understanding or independently completing the questionnaire were also excluded. These criteria were assessed through self-report screening questions at the beginning of the survey rather than through clinical examination or formal cognitive assessment.

Data were collected through an online survey administered using Wenjuanxing (Questionnaire Star; Changsha Ranxing Information Technology Co., Ltd., China). Participants accessed the questionnaire through a survey link or QR code distributed at the participating universities. Before beginning the survey, participants were presented with an electronic information sheet describing the study purpose, voluntary nature of participation, confidentiality of the data, and their right to discontinue participation at any time. Participants provided electronic informed consent before proceeding to the questionnaire. No directly identifying information, such as participants’ names or student identification numbers, was requested. Survey responses were analyzed using study codes rather than personal identifiers, and the research dataset contained no information that allowed individual participants to be directly identified.

Data quality screening was conducted before analysis. Responses were excluded if they were incomplete, showed careless or patterned responding (e.g., invariant responses across items), or were completed in less than 180 s, which was set as the minimum acceptable completion time. No participants were excluded based solely on statistical outlier detection.

For evaluation of the factor structure of the Chinese version of the Physical Effort Scale (PES), the final sample was randomly divided into two independent subsamples using SPSS: 653 participants for exploratory factor analysis (EFA) and 620 participants for confirmatory factor analysis (CFA). Gender and academic year were not used as stratification variables during randomization. Comparisons between the two subsamples showed no significant differences in gender distribution, χ^2^(1) = 0.000, p = 0.985, or academic-year distribution, χ^2^(3) = 0.083, p = 0.994.

### 2.2 Instrument

#### 2.2.1 Physical Effort Scale (PES)

Tendencies to approach and avoid physical effort were assessed using the Chinese version of the Physical Effort Scale (PES), which was translated and culturally adapted in the present study based on the original scale developed by Cheval et al. (2024). The PES includes eight items across two dimensions: physical effort approach and physical effort avoidance. Items are rated on a 5-point scale from 1 (strongly disagree) to 5 (strongly agree), with higher scores indicating stronger approach or avoidance tendencies. The translation and cultural adaptation procedures are described in Section 2.3.

#### 2.2.2 Behavioral Regulation in Exercise Questionnaire–3 (BREQ-3)

Exercise motivation was measured using the Chinese version of the BREQ-3 (Luo et al., 2022). The 24-item scale assesses six types of behavioral regulation on a 5-point scale ranging from 1 (not true for me) to 5 (very true for me). The present study used scores for amotivation, external regulation, introjected regulation, and autonomous motivation. Autonomous motivation was calculated from identified, integrated, and intrinsic regulation items. Item 19 was excluded based on previous validation findings showing weak loading on autonomous motivation (Luo et al., 2022).

#### 2.2.3 International Physical Activity Questionnaire–Short Form (IPAQ-SF)

Physical activity was assessed using the IPAQ-SF (Craig et al., 2003), which measures vigorous-intensity activity, moderate-intensity activity, walking, and sedentary behavior during the previous 7 days. Data were processed according to the standard IPAQ scoring protocol. Durations below 10 min were recoded as zero, and activity durations exceeding 180 min/day were truncated. Responses with total reported activity exceeding 960 min/day were treated as implausible and excluded from the corresponding analyses. Total physical activity was calculated as MET-min/week and was used as the primary indicator of overall physical activity volume. In addition, moderate-to-vigorous physical activity (MVPA) was derived from the moderate- and vigorous-intensity activity data and was examined as a supplementary indicator to facilitate comparison with previous PES validation studies.

#### 2.2.4 Weekly exercise frequency

Weekly exercise frequency was assessed using a single item: “How many times do you exercise per week?” Response options were 0, 1–2, 3–4, and ≥5 times per week, coded as 0, 1, 2, and 3, respectively, with higher scores indicating greater weekly exercise frequency. This measure was assessed separately from the IPAQ-derived total physical activity (MET-min/week).

#### 2.2.5 Emotion Regulation Questionnaire (ERQ)

Emotion regulation was assessed using the Chinese version of the ERQ (Wang et al., 2007). The 10-item scale measures cognitive reappraisal and expressive suppression using a 7-point scale from 1 (strongly disagree) to 7 (strongly agree), with higher scores indicating greater use of each strategy.

### 2.3 Translation and cross-cultural adaptation

The original Physical Effort Scale (PES) developed by Cheval et al. (2024) was translated and culturally adapted into Chinese with permission from the original authors (see Supplementary Figure 1 and Supplementary Table 1). The procedure was conducted with reference to Beaton et al. (2000). Two bilingual translators independently conducted the forward translation: one was a doctoral student in sport science (Z.Q.), and the other was a master’s student in English studies (T.A.Q.). The two translations were compared and synthesized into a preliminary Chinese version, with discrepancies resolved through discussion.

The preliminary version was back-translated into English by two independent bilingual translators: a master’s student in sport science (H.P.S.H.) and a master’s student in English studies (P.X.F.). An expert panel consisting of three sport psychology experts, one psychometrician, and one linguistics expert reviewed the Chinese version and the back-translated version against the original PES, focusing on semantic, conceptual, and cultural equivalence.

The pre-final version was pilot tested in 173 university students, of whom 157 provided valid responses. After completing the questionnaire, participants provided feedback on item clarity and comprehensibility. Based on participant feedback and item-level analyses, minor wording adjustments were made before finalizing the Chinese PES. Test–retest reliability was assessed in 106 participants who completed the PES at two time points; these data were not included in the main validation analyses.

### 2.4 Procedure

Data were collected through an online survey between April and May 2026. Before completing the questionnaire, all participants provided electronic informed consent. Participation was voluntary and anonymous. The study was approved by the Ethics Committee of Nanchang University (Approval No. NCUREC202604012) and was conducted in accordance with the ethical principles of the Declaration of Helsinki.

### 2.5 Statistical analysis

All analyses were conducted using IBM SPSS Statistics 27.0, R 4.6.1, and Mplus 8.3. Descriptive statistics were calculated for all study variables. Item performance was examined using corrected item–total correlations, critical-ratio tests, and Cronbach’s α if item deleted. Reliability was evaluated using Cronbach’s α, McDonald’s ω, and composite reliability. Test–retest reliability was assessed using the single-measure intraclass correlation coefficient (ICC) based on a two-way mixed-effects model with absolute agreement.

The factor structure of the Chinese Physical Effort Scale (PES) was examined using exploratory factor analysis (EFA) and confirmatory factor analysis (CFA) in independent subsamples. EFA was conducted using principal axis factoring with direct oblimin rotation. CFA was estimated using the weighted least squares mean- and variance-adjusted estimator with theta parameterization, treating PES items as ordered categorical indicators. Model fit was evaluated using the comparative fit index (CFI), Tucker–Lewis index (TLI), root mean square error of approximation (RMSEA), and standardized root mean square residual (SRMR). Following commonly used guidelines, CFI and TLI values close to or above 0.95, RMSEA values close to or below 0.06, and SRMR values close to or below 0.08 were considered indicative of good fit (Hu and Bentler, 1999). These values were treated as guidelines rather than strict cutoff criteria, and model fit was evaluated across multiple indices. Given that RMSEA can be sensitive to small model degrees of freedom, it was interpreted alongside CFI, TLI, and SRMR rather than as a standalone indicator of model fit (Kenny et al., 2015). Convergent validity was evaluated using standardized factor loadings, composite reliability (CR), and average variance extracted (AVE), with CR values of 0.70 or higher and AVE values of 0.50 or higher used as reference criteria (Fornell and Larcker, 1981). Discriminant validity was examined using the latent factor correlation and the heterotrait–monotrait ratio (HTMT). McDonald’s ω was estimated as an additional measure of scale-score reliability rather than as an indicator of convergent validity (Hayes and Coutts, 2020).

Measurement invariance across gender and academic year was tested sequentially at the configural, metric, and scalar levels. The correlated residuals between PES5–PES6 and PES7–PES8 specified in the final CFA model were retained in all multigroup models. Invariance was evaluated using changes in CFI, RMSEA, and SRMR, together with WLSMV-based model comparisons (Vandenberg and Lance, 2000). Criterion-related validity was assessed using Pearson correlations between PES dimensions and exercise motivation and emotion regulation. Spearman correlations were used for Total MET-min/week and MVPA because of their non-normal distributions, and differences in PES scores across physical activity levels were examined using the Kruskal–Wallis H test.

Latent profile analysis (LPA) was conducted in the full sample using physical effort approach and avoidance scores as profile indicators (see Supplementary Appendix 1). The primary model constrained indicator variances to be equal across profiles and fixed the within-profile covariance between the two indicators to zero. Models with one to six profiles were estimated, and profile selection was guided by AIC, BIC, aBIC, entropy, the LMR-LRT, BLRT, profile size, and theoretical interpretability (Nylund et al., 2007). Classification quality was further assessed using average posterior probabilities and estimated versus most-likely class proportions. Sensitivity analyses examined increased random starts, class-specific indicator variances, and a freely estimated common within-profile covariance (see Supplementary Appendices 2–4). Distal psychological and behavioral outcomes were compared across profiles using the BCH approach to account for classification uncertainty (Bakk and Vermunt, 2016). All tests were two-tailed, with statistical significance set at *p* < 0.05.

## 3 Results

### 3.1 Participant characteristics

Participant characteristics are summarized in Table 1. The final sample included 1,273 university students, with 630 males (49.5%) and 643 females (50.5%). Participants were almost evenly distributed across academic grades, and students from six universities in different regions of China were represented.

**TABLE 1:** Sample characteristics (*N* = 1,273).

Variable	Category	*n*	%
Total sample		1273	100
Gender	Male	630	49.5
	Female	643	50.5
Grade	Freshman	312	24.5
	Sophomore	321	25.2
	Junior	322	25.3
	Senior	318	25.0
Major type	Physical education majors	225	17.7
	Non-physical education majors	1048	82.3

Descriptive statistics for the study variables are presented in Table 2. Participants reported moderate levels of physical effort approach and avoidance. Exercise motivation scores indicated relatively higher autonomous motivation than non-autonomous forms of motivation. Skewness and kurtosis values for all variables fell within acceptable ranges, suggesting no substantial departure from normality.

**TABLE 2:** Descriptive statistics of the study variables (*N* = 1,273).

Dimension	Items (*k*)	Mean (*M*)	*SD*	Skewness	Kurtosis
PES-Approach	4	3.253	1.010	−0.103	−0.483
PES-Avoidance	4	3.031	0.997	−0.075	−0.449
ERQ-Reappraisal	6	5.275	1.125	−0.352	−0.273
ERQ-Suppression	4	5.047	1.228	−0.366	−0.153
BREQ-3-AM	4	3.137	1.028	−0.095	−0.544
BREQ-3-ER	4	2.947	1.002	0.080	−0.579
BREQ-3-IR	4	2.893	0.985	0.129	−0.500
BREQ-3-AUT	11	3.318	0.896	−0.399	−0.106

PES, Physical Effort Scale; ERQ, Emotion Regulation Questionnaire; BREQ-3, Behavioral Regulation in Exercise Questionnaire-3; AM, amotivation; ER, external regulation; IR, introjected regulation; AUT, autonomous regulation; k, number of items; M, mean; SD, standard deviation.

### 3.2 Psychometric properties of the Chinese PES

#### 3.2.1 Item analysis and reliability

In the pilot sample (*N* = 157), Cronbach’s α coefficients for the subscales ranged from 0.766 to 0.945. Principal axis factoring with Promax rotation yielded two-factor solutions for the PES and ERQ, accounting for 78.47% and 73.10% of the variance after extraction, respectively. For the revised Chinese BREQ-3, the specified four-factor solution accounted for 58.36% of the variance after extraction. Internal-consistency reliability results for the pilot sample are presented in Supplementary Table 2, and detailed exploratory factor analysis results are provided in Supplementary Tables 3–5.

In the main sample, Cronbach’s α coefficients were 0.935 for the Approach dimension and 0.913 for the Avoidance dimension, both exceeding the commonly used 0.70 criterion for adequate internal consistency (Nunnally and Bernstein, 1994). Test–retest reliability was examined in 106 participants who completed the PES at two time points. The single-measure ICC was 0.794 (95% CI [0.709, 0.856], *p* < 0.001); the point estimate falls within the range commonly classified as good reliability, although the confidence interval extends from the moderate to good range (Koo and Li, 2016). Cronbach’s α coefficients for the ERQ and BREQ-3 dimensions ranged from 0.766 to 0.935 and also exceeded the 0.70 criterion. Reliability results for the PES are presented in Table 3.

**TABLE 3:** Internal consistency reliability of the Chinese version of the Physical Effort Scale (PES) (*N* = 1,273).

Scale	Dimension	Items (*k*)	Cronbach’s α
PES	Approach	4	0.935
PES	Avoidance	4	0.913

*k*, number of items.

Item-analysis results are presented in Table 4. Extreme-group comparisons were significant for all eight PES items (all *p* < 0.001), and corrected item–total correlations ranged from 0.769 to 0.856. In addition, Cronbach’s α after deletion of any individual item was lower than the overall α coefficient of the corresponding dimension. Based on these results, all eight items were retained for subsequent analyses.

**TABLE 4:** Item analysis of the Chinese version of the Physical Effort Scale (PES) (*N* = 1,273).

Dimension	Item	CR (*t*)	CITC	Cronbach’s α if item deleted
Approach	PES1	47.300***	0.839	0.918
	PES2	50.819***	0.856	0.912
	PES3	52.121***	0.856	0.912
	PES4	48.290***	0.835	0.919
Avoidance	PES5	42.797***	0.769	0.899
	PES6	48.518***	0.819	0.882
	PES7	48.481***	0.800	0.888
	PES8	52.164***	0.820	0.881

CR, critical ratio; CITC, corrected item–total correlation.

***p < 0.001.

#### 3.2.2 Exploratory factor analysis

In the exploratory factor analysis sample (*n* = 653), the suitability of the data for factor analysis was first assessed. The Kaiser–Meyer–Olkin (KMO) measure was 0.919, and Bartlett’s test of sphericity was statistically significant (χ^2^ = 4027.354, df = 28, *p* < 0.001), indicating that the data were well suited for factor analysis. Exploratory factor analysis was subsequently conducted on the eight PES items using principal axis factoring (PAF) with Direct Oblimin oblique rotation. Based on the eigenvalue-greater-than-one criterion and inspection of the scree plot, two common factors were extracted, accounting for 80.10% of the total variance. The standardized factor loadings ranged from 0.807 to 0.890, and all items loaded on their theoretically corresponding factors, with no substantial cross-loadings observed. The two factors showed a moderately strong negative correlation (*r* = −0.699), supporting the two-factor structure of the Chinese version of the PES.

#### 3.2.3 Confirmatory factor analysis

Confirmatory factor analysis was conducted to examine the two-factor structure of the PES, with the results presented in Table 5. Based on the modification indices and item content, correlated residuals were permitted only between PES5 and PES6 and between PES7 and PES8. PES5 and PES6 both concern avoidance of situations or tasks involving physical effort, whereas PES7 and PES8 involve negative evaluations of physical activity; therefore, the residual correlations may reflect local dependence associated with similarities in item content. The final model yielded χ^2^(17) = 165.532, *p* < 0.001, CFI = 0.996, TLI = 0.994, RMSEA = 0.119, 90% CI [0.103, 0.136], and SRMR = 0.016. CFI, TLI, and SRMR met the commonly used fit guidelines described above, whereas RMSEA substantially exceeded the corresponding benchmark. Although RMSEA can be inflated in models with small degrees of freedom (Kenny et al., 2015), the elevated value indicates that the overall model fit should be interpreted cautiously.

**TABLE 5:** Confirmatory factor analysis of the Chinese version of the Physical Effort Scale (PES) (*n* = 620).

Factor	Items	Loading range	Composite reliability	AVE	McDonald’s ω
Approach	4	0.908–0.977	0.971	0.895	0.971
Avoidance	4	0.801–0.913	0.926	0.759	0.926

AVE, average variance extracted.

Standardized factor loadings ranged from 0.801 to 0.977. Composite reliability (CR) and average variance extracted (AVE) exceeded the commonly used criteria of 0.70 and 0.50, respectively, providing evidence relevant to convergent validity (Fornell and Larcker, 1981). McDonald’s ω was reported separately as an additional estimate of scale-score reliability rather than as an indicator of convergent validity (Hayes and Coutts, 2020). The standardized latent correlation between the Approach and Avoidance factors was −0.469 (*p* < 0.001), and the HTMT value was 0.441.

#### 3.2.4 Common method bias

Because all major variables were assessed using self-report questionnaires, potential common method bias was examined. Harman’s single-factor test showed that the first unrotated factor accounted for 59.606% of the total variance. Given the limitations of this approach and the absence of a latent method factor model, this result was considered only as a preliminary assessment, and potential common method bias could not be completely excluded.

### 3.3 Measurement invariance

#### 3.3.1 Measurement invariance across gender

Measurement invariance across gender was examined using multigroup confirmatory factor analysis, with the correlated residuals between PES5–PES6 and PES7–PES8 retained in all groups as specified in the final CFA model (see Table 6). The configural model showed good fit across most indices, although the RMSEA was relatively high, suggesting that the absolute fit should be interpreted with caution. Imposing metric and scalar invariance constraints did not result in substantial deterioration of model fit (ΔCFI ≤ 0.001, ΔRMSEA ≤ 0, ΔSRMR ≤ 0.003), and WLSMV-based model comparisons were nonsignificant (*p*s > 0.05). Overall, the findings support metric and scalar invariance of the Chinese version of the PES across gender, although this conclusion should be considered in light of the elevated RMSEA in the configural model.

**TABLE 6:** Measurement invariance across gender (*n* = 620).

Model	χ^2^ (df)	CFI	TLI	RMSEA [90% CI]	SRMR
Configural	137.740 (34)	0.997	0.995	0.099 [0.082, 0.117]	0.017
Metric	133.464 (40)	0.997	0.996	0.087 [0.071, 0.103]	0.018
Scalar	121.344 (62)	0.998	0.998	0.056 [0.041, 0.070]	0.021

CFI, comparative fit index; TLI, Tucker–Lewis index; RMSEA, root mean square error of approximation; CI, confidence interval; SRMR, standardized root mean square residual; df, degrees of freedom.

#### 3.3.2 Measurement invariance across academic year groups

Measurement invariance of the Chinese version of the PES across academic years was further examined, with the two correlated residuals retained from the final CFA model in all groups (see Table 7). The configural model showed acceptable fit across most indices, although the elevated RMSEA suggested some caution in interpreting absolute model fit. Imposing metric and scalar invariance constraints did not result in meaningful deterioration in model fit, and WLSMV-based model comparisons were nonsignificant (*p*s > 0.05). These findings suggest that metric and scalar invariance were supported across academic years; however, this conclusion should be interpreted cautiously given the relatively high RMSEA of the configural model.

**TABLE 7:** Measurement invariance across academic years (*n* = 620).

Model	χ^2^ (*df*)	CFI	TLI	RMSEA [90% CI]	SRMR
Configural	264.698 (68)	0.995	0.992	0.137 [0.119, 0.154]	0.024
Metric	264.410 (86)	0.996	0.995	0.116 [0.100, 0.132]	0.024
Scalar	244.520 (152)	0.998	0.998	0.063 [0.048, 0.077]	0.028

CFI, comparative fit index; TLI, Tucker–Lewis index; RMSEA, root mean square error of approximation; CI, confidence interval; SRMR, standardized root mean square residual; *df*, degrees of freedom.

### 3.4 Criterion-related validity

#### 3.4.1 Associations with psychological variables

To further examine the construct validity of the Chinese version of the Physical Effort Scale (PES), correlations with theoretically related constructs were examined. As shown in Table 8, PES-Approach was negatively associated with PES-Avoidance, and the two dimensions showed different patterns of associations with emotion regulation and exercise motivation. PES-Approach was positively associated with cognitive reappraisal, expressive suppression, external regulation, introjected regulation, and autonomous regulation, and negatively associated with amotivation. PES-Avoidance showed the opposite pattern, being negatively associated with these emotion-regulation and motivational variables and positively associated with amotivation. These findings indicate systematic but distinct associations of the two PES dimensions with emotion regulation and exercise motivation.

**TABLE 8:** Correlations among the study variables (*N* = 1,273).

Variables	*M*	*SD*	1	2	3	4	5	6	7	8
1. PES-Approach	3.253	1.010	–	–	–	–	–	–	–	–
2. PES-Avoidance	3.031	0.997	−0.552**							
3. ERQ-Reappraisal	5.275	1.125	0.485**	−0.427**						
4. ERQ-Suppression	5.047	1.228	0.346**	−0.276**	0.682**					
5. BREQ-AM	3.137	1.028	−0.286**	0.176**	−0.227**	−0.372**				
6. BREQ-ER	2.947	1.002	0.338**	−0.196**	0.278**	0.381**	−0.838**			
7. BREQ-IR	2.893	0.985	0.406**	−0.256**	0.285**	0.396**	−0.814**	0.839**		
8. BREQ-AUT	3.318	0.896	0.664**	−0.520**	0.588**	0.431**	−0.453**	0.545**	0.594**	

PES, Physical Effort Scale; ERQ, Emotion Regulation Questionnaire; BREQ-3, Behavioral Regulation in Exercise Questionnaire-3; AM, amotivation; ER, external regulation; IR, introjected regulation; AUT, autonomous regulation; *M*, mean; *SD*, standard deviation. Values are Pearson’s correlation coefficients.

*p < 0.05,

**p < 0.01.

#### 3.4.2 Associations with physical activity

To further evaluate the external validity of the Chinese version of the PES, associations with physical activity were examined using the International Physical Activity Questionnaire–Short Form (IPAQ-SF). Because Total MET-min/week and MVPA were nonnormally distributed, Spearman rank correlations were used. PES-Approach was positively associated with Total MET-min/week (ρ = 0.153, *p* < 0.001) and MVPA (ρ = 0.170, *p* < 0.001), whereas PES-Avoidance was negatively associated with Total MET-min/week (ρ = −0.114, *p* < 0.001) and MVPA (ρ = −0.113, *p* < 0.001).

Participants were further classified into low, moderate, and high physical activity groups based on IPAQ-SF criteria. Kruskal–Wallis tests revealed significant group differences in PES-Approach (*H* = 25.355, *p* < 0.001) and PES-Avoidance (*H* = 6.333, *p* = 0.042). Notably, individuals with high physical activity levels reported lower avoidance tendencies, providing additional support for the external validity of the Chinese PES.

### 3.5 Physical-effort approach and avoidance

#### 3.5.1 Model comparison

Latent profile analyses were conducted by estimating one- to six-profile models (Table 9). AIC, BIC, and adjusted BIC decreased as the number of profiles increased, and both the LMR-LRT and BLRT remained significant across the two- to six-profile solutions. However, the four- to six-profile solutions contained very small classes (0.31%–1.41%), whereas the smallest class in the three-profile solution represented 14.93% of the sample. Considering this pattern together with profile interpretability, the three-profile solution was retained (entropy = 0.770). The three profiles were labeled the Balanced Profile (59.07%), Avoidance-Oriented Profile (14.93%), and Approach-Oriented Profile (26.00%).

**TABLE 9:** Model fit indices for latent profile analysis (*N* = 1,273).

Classes	AIC	BIC	aBIC	Entropy	LMR-LRT *p*	BLRT *p*	Smallest class (%)
1	7248.679	7269.276	7256.570	–	–	–	100.00
2	6919.227	6955.271	6933.036	0.592	<0.001	<0.001	35.66
**3**	**6738.591**	**6790.082**	**6758.318**	**0.770**	**<0.001**	**<0.001**	**14.93**
4	6643.834	6710.773	6669.479	0.820	<0.001	<0.001	1.34
5	6606.066	6688.452	6637.628	0.804	0.0007	<0.001	1.41
6	6576.549	6674.382	6614.029	0.819	<0.001	<0.001	0.31

AIC, Akaike Information Criterion; BIC, Bayesian Information Criterion; aBIC, Sample-size Adjusted BIC; LMR, Lo–Mendell–Rubin Likelihood Ratio Test; BLRT, Bootstrap Likelihood Ratio Test. Bold indicates the final selected model.

For the three-profile solution, average posterior probabilities for the most likely class membership ranged from 0.888 to 0.907. Estimated class proportions were similar to those based on most likely class assignment. Additional classification diagnostics and sensitivity analyses are reported in the Supplementary Tables 6–10.

#### 3.5.2 Profile characteristics

Based on the three-profile solution, participants were classified into the Balanced (59.07%, *n* = 752), Avoidance-Oriented (14.93%, *n* = 190), and Approach-Oriented (26.00%, *n* = 331) profiles (Figure 1 and Table 10). The profiles differed in their relative levels of physical-effort approach and avoidance. Profile membership differed by academic major, χ^2^(2) = 50.98, *p* < 0.001, Cramér’s *V* = 0.20. Based on classifications from the full-sample solution, 43.6% of physical education students were classified as Approach-Oriented, 50.7% as Balanced, and 5.8% as Avoidance-Oriented, compared with 22.2%, 60.9%, and 16.9%, respectively, among students from other majors.

**FIGURE 1:**
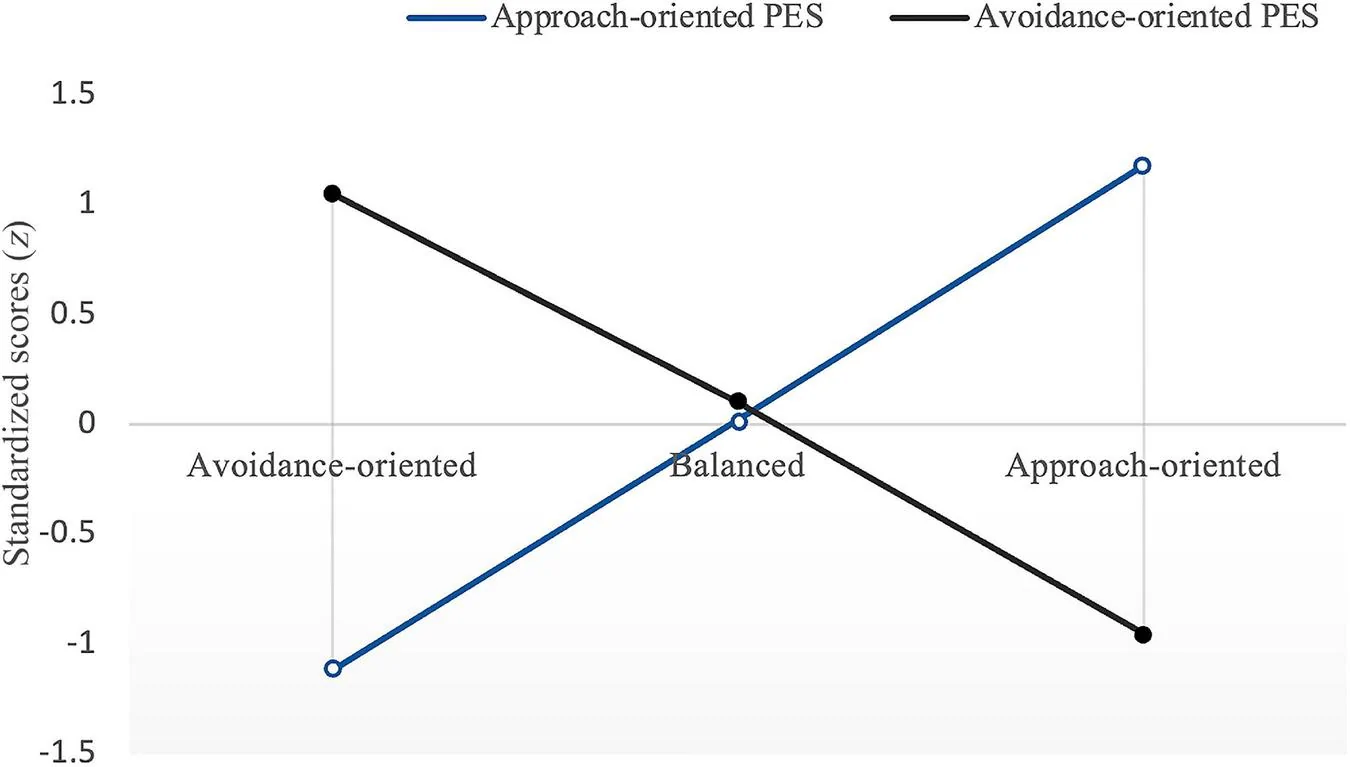
Latent profiles based on standardized scores for the approach and avoidance dimensions of the Physical Effort Scale (PES).

**TABLE 10:** Characteristics of the latent profiles of physical-effort approach and avoidance (*N* = 1,273).

Class	*n*	Percentage (%)	Approach	Avoidance	Profile name
Class1	752	59.07	3.107	3.140	Balanced
Class2	190	14.93	1.753	4.028	Avoidance-Oriented
Class3	331	26.00	4.452	2.209	Approach-Oriented

PES, Physical Effort Scale. Approach and Avoidance values represent mean subscale scores.

#### 3.5.3 Differences in physical activity levels and weekly exercise frequency across latent profiles

Bolck–Croon–Hagenaars analyses showed differences in weekly exercise frequency across the three profiles, χ^2^(2) = 6.331, *p* = 0.042 (Table 11). The Approach-Oriented profile reported higher weekly exercise frequency than the Balanced profile (*p* = 0.016), whereas the other pairwise differences were not significant (*p*s ≥ 0.099).

**TABLE 11:** Differences in physical activity behaviors across latent profiles (*N* = 1273).

Variable	Avoidance-Oriented	Balanced	Approach-Oriented	χ^2^	*p*
Weekly exercise frequency	1.965	1.945	2.105	6.331	0.042
Total MET-min/week	1966.57	2011.18	2567.50	24.145	<0.001
MVPA (MET-min/week)	970.89	1032.09	1503.91	29.107	<0.001

MET-min/week, metabolic equivalent minutes per week. Values represent profile-specific means.

Total physical activity (MET-min/week) differed across profiles, χ^2^(2) = 24.145, *p* < 0.001. The Approach-Oriented profile had higher total physical activity than both the Balanced and Avoidance-Oriented profiles (both *p*s < 0.001), whereas the latter two did not differ (*p* = 0.719). A similar pattern was observed for MVPA, χ^2^(2) = 29.107, *p* < 0.001. The Approach-Oriented profile had higher MVPA than both the Balanced and Avoidance-Oriented profiles (both *p*s < 0.001), whereas the latter two did not differ (*p* = 0.506).

Sensitivity analyses excluding physical education students (*n* = 225) yielded the same pattern among the remaining 1,048 participants. The overall difference in total physical activity remained significant, χ^2^(2) = 22.080, *p* < 0.001, with the Approach-Oriented profile reporting higher total physical activity than both the Balanced and Avoidance-Oriented profiles (both *p*s < 0.001), whereas the latter two did not differ (*p* = 0.828). Similarly, the overall difference in MVPA remained significant, χ^2^(2) = 25.620, *p* < 0.001. The Approach-Oriented profile reported higher MVPA than both the Balanced and Avoidance-Oriented profiles (both *p*s < 0.001), whereas the Balanced and Avoidance-Oriented profiles did not differ (*p* = 0.778); see Supplementary Table 11.

#### 3.5.4 Differences in external variables

Bolck–Croon–Hagenaars analyses were conducted to compare the three latent profiles on external variables, including emotion regulation strategies, exercise motivation, and physical activity levels (Table 12). Significant differences were observed across all external indicators (all *p*s < 0.001).

**TABLE 12:** Comparisons of emotion regulation and exercise motivation across latent profiles using the BCH method (*N* = 1273).

Variable	Avoidance-Oriented	Balanced	Approach-Oriented	χ^2^	*p*
ERQ_Reappraisal	4.242	5.172	6.107	332.865	<0.001
ERQ_Suppression	4.344	4.941	5.690	117.632	<0.001
BREQ_AM	3.747	3.133	2.777	83.566	<0.001
BREQ_ER	2.301	2.910	3.410	107.153	<0.001
BREQ_IR	2.155	2.853	3.419	170.259	<0.001
BREQ_AUT	2.162	3.255	4.140	720.600	<0.001

BCH, Bolck–Croon–Hagenaars; ERQ, Emotion Regulation Questionnaire; BREQ-3, Behavioral Regulation in Exercise Questionnaire-3; AM, amotivation; ER, external regulation; IR, introjected regulation; AUT, autonomous regulation. Values represent profile-specific estimated means.

For emotion regulation, the Approach-Oriented Profile showed the highest levels of cognitive reappraisal and expressive suppression, followed by the Balanced Profile and the Avoidance-Oriented Profile, with all pairwise comparisons reaching significance (all *p*s < 0.001). Mean cognitive reappraisal scores were 6.107, 5.172, and 4.242 for the Approach-Oriented, Balanced, and Avoidance-Oriented Profiles, respectively.

Exercise motivation also differed significantly across profiles. The Approach-Oriented Profile demonstrated significantly higher levels of external regulation, introjected regulation, and autonomous regulation than the Balanced and Avoidance-Oriented Profiles, whereas the Balanced Profile scored higher than the Avoidance-Oriented Profile on these dimensions (all *p*s < 0.001). In contrast, amotivation followed the opposite pattern, with the Avoidance-Oriented Profile reporting the highest level of amotivation, followed by the Balanced Profile, whereas the Approach-Oriented Profile showed the lowest level (all *p*s < 0.001).

## 4 Discussion

### 4.1 Summary of main findings

This study evaluated the psychometric properties of the Chinese version of the Physical Effort Scale (PES) and examined individual differences in approach and avoidance tendencies toward physical effort among Chinese university students, together with their associations with emotion regulation, exercise motivation, and physical activity. The PES conceptualizes physical-effort approach and avoidance as related but distinguishable tendencies (Cheval et al., 2024). The EFA supported a two-factor structure comprising physical-effort approach and physical-effort avoidance. In the CFA, CFI, TLI, and SRMR indicated good fit, whereas RMSEA remained elevated, indicating mixed evidence regarding overall model fit. Therefore, the overall model fit should be interpreted with caution. In the measurement invariance analyses, imposing metric and scalar constraints across gender and academic-year groups resulted in little deterioration in model fit. These findings were consistent with metric and scalar invariance across the groups examined, although they should be interpreted cautiously given the elevated RMSEA values of the configural models.

For the person-centered analysis, a three-profile solution was retained in the current sample, comprising the Balanced, Avoidance-Oriented, and Approach-Oriented Profiles. The profiles were largely ordered along a continuum from low approach–high avoidance to high approach–low avoidance. Thus, the observed pattern may substantially reflect a continuous approach–avoidance gradient rather than qualitatively distinct, naturally occurring subgroups. Accordingly, the profiles are better interpreted as descriptive configurations of the joint distribution of the two PES dimensions.

Bolck–Croon–Hagenaars analyses further showed differences across the three profiles in emotion regulation, exercise motivation, and physical activity. The Approach-Oriented Profile showed higher levels of both cognitive reappraisal and expressive suppression, together with higher external regulation, introjected regulation, and autonomous regulation and lower amotivation. This mixed pattern does not support characterizing the Approach-Oriented Profile as globally more adaptive. This interpretation is consistent with the distinction between autonomous and controlled forms of motivation emphasized in self-determination theory (Ryan and Deci, 2000; Teixeira et al., 2012). With respect to physical activity, the Approach-Oriented Profile reported higher total physical activity than the Balanced and Avoidance-Oriented Profiles, whereas no significant difference was observed between the latter two profiles. Overall, the findings indicate that different configurations of physical-effort approach and avoidance were associated with differences in emotion regulation, exercise motivation, and physical activity in the current sample.

### 4.2 Psychometric properties of the Chinese PES

The present study provides preliminary evidence regarding the psychometric properties of the Chinese version of the PES among university students. The EFA supported the expected two-factor structure comprising physical-effort approach and physical-effort avoidance, whereas the CFA provided mixed evidence regarding overall model fit. CFI, TLI, and SRMR indicated good fit, whereas RMSEA remained elevated. Although RMSEA can be unstable in models with low degrees of freedom (Kenny et al., 2015), its elevated value should not be disregarded. The latent factor correlation and HTMT value were also consistent with the empirical distinguishability of the two dimensions. Both subscales showed high internal consistency, and the test–retest results indicated good temporal reliability. However, the need to allow correlated residuals between PES5–PES6 and PES7–PES8 suggests possible local dependence related to overlapping item content. Accordingly, the internal structure of the Chinese PES should be further examined in independent samples. These findings are broadly consistent with the conceptualization of physical-effort approach and avoidance as related but distinguishable tendencies (Cheval et al., 2024). Broader theoretical accounts of subjective effort describe bodily, cognitive, affective, and motivational processes as relevant to effort-related experiences (Alvarez-Alvarado and Tenenbaum, 2021; Welch et al., 2010; de la Garanderie et al., 2023; Halperin and Vigotsky, 2024; Herzberg et al., 2026; Pageaux, 2016). These perspectives provide a broader theoretical context but should not be taken to imply that the PES directly measures these underlying processes.

In the multigroup analyses across gender and academic year, imposing metric and scalar invariance constraints did not result in substantial deterioration in model fit. These results were consistent with metric and scalar invariance across the gender and academic-year groups examined. However, the RMSEA values of the configural models remained elevated, particularly across academic-year groups, so the invariance findings should be interpreted cautiously. Although the translation and adaptation of the Chinese PES followed established principles of cross-cultural scale adaptation (Beaton et al., 2000), the present study did not include comparisons across cultural or linguistic groups. Accordingly, the findings should not be interpreted as evidence of cross-cultural measurement invariance.

### 4.3 Latent profiles of physical-effort approach and avoidance and their psychological and behavioral correlates

Although a three-profile solution was retained in the current sample, the profiles were largely ordered from low approach–high avoidance, through moderate approach–moderate avoidance, to high approach–low avoidance. Accordingly, these profiles are more appropriately interpreted as empirical configurations of the joint distribution of the two PES dimensions rather than as qualitatively distinct, naturally occurring subgroups. Nevertheless, the person-centered analysis provides a complementary perspective on how physical-effort approach and avoidance tendencies co-occur within individuals. Broader theoretical accounts describe subjective effort as involving physiological, cognitive, affective, motivational, and contextual processes (Ekkekakis, 2003; Marcora, 2009; Parfitt and Hughes, 2009; Abbiss et al., 2015; Pageaux, 2016; Halperin and Vigotsky, 2024). However, the PES specifically assesses individual differences in approach and avoidance tendencies toward physical effort (Cheval et al., 2024), and these broader processes were not directly assessed in the present study.

The profiles were associated with different patterns of exercise motivation. The Approach-Oriented Profile showed the highest autonomous regulation and the lowest amotivation, but it also showed the highest external and introjected regulation, whereas the Balanced Profile generally showed intermediate levels. This combination indicates a mixed motivational pattern rather than a uniformly more autonomous or more adaptive profile. This pattern can be considered in relation to self-determination theory, which distinguishes autonomous from controlled forms of motivation and emphasizes the broader relevance of motivational regulation to health-related behavior (Ryan and Deci, 2000; Teixeira et al., 2012; Vansteenkiste et al., 2020; Ntoumanis et al., 2021). The present findings indicate that the high approach–low avoidance configuration was associated with higher levels of both autonomous and controlled forms of regulation. However, the cross-sectional design does not establish temporal or directional relationships between physical-effort tendencies and motivational regulation.

A similarly mixed pattern was observed for emotion regulation. The Approach-Oriented Profile reported the highest levels of both cognitive reappraisal and expressive suppression. Cognitive reappraisal and expressive suppression represent distinct emotion-regulation strategies (Gross and John, 2003), and emotion-regulation processes have also been considered in exercise-related contexts (Gürdere et al., 2024). Their simultaneous elevation in the present study does not support a uniformly more adaptive interpretation of emotion regulation in the Approach-Oriented Profile. Rather, the findings indicate greater reported use of both strategies among participants in the high approach–low avoidance configuration, without establishing whether this combination is beneficial or detrimental.

With respect to self-reported physical activity, the Approach-Oriented Profile reported a higher weekly exercise frequency than the Balanced Profile, whereas its difference from the Avoidance-Oriented Profile was not statistically significant. Both Total MET-min/week and MVPA were higher in the Approach-Oriented Profile than in the Balanced and Avoidance-Oriented Profiles, whereas the latter two profiles did not differ on either measure. Thus, although the Balanced and Avoidance-Oriented Profiles differed substantially in their configurations of physical-effort approach and avoidance, these differences were not accompanied by detectable differences in overall physical activity or MVPA. In contrast, the high approach–low avoidance configuration was consistently associated with higher activity levels in the current sample. Profile membership also differed by academic major, but the main pattern for total physical activity remained after excluding physical education students. Previous cross-sectional research among Chinese university students has likewise reported a positive association between exercise motivation and physical activity (Zhang et al., 2024). These findings can be considered within broader models in which physical activity is related to multiple motivational, affective, and cognitive factors (Bandura, 2004; Brand and Ekkekakis, 2018; Rhodes et al., 2019; Gerber et al., 2025). More specifically, the Theory of Effort Minimization in Physical Activity proposes that effort-related aversion may contribute to tendencies to minimize physical effort (Cheval and Boisgontier, 2021). These perspectives provide interpretive context rather than mechanisms demonstrated by the present data, and the observed differences should not be attributed to any single psychological process.

Taken together, the findings indicate that different configurations of physical-effort approach and avoidance were associated with different patterns of exercise motivation, emotion regulation, and physical activity. The profiles should therefore be regarded primarily as descriptive and hypothesis-generating configurations rather than as established intervention targets.

### 4.4 Limitations and future directions

Several limitations should be acknowledged. First, the cross-sectional design precludes conclusions about temporal ordering or causality among physical-effort approach and avoidance, exercise motivation, emotion regulation, and physical activity. Although the Chinese PES demonstrated good short-term test–retest reliability, the longitudinal stability of the latent profiles remains unknown. Second, the measurement structure warrants further examination because RMSEA remained elevated in the final CFA model and correlated residuals between PES5–PES6 and PES7–PES8 suggested possible local dependence, although RMSEA may be unstable in models with low degrees of freedom (Kenny et al., 2015). Third, the study relied primarily on self-report measures and may therefore be affected by recall bias, social desirability, and common method bias. Fourth, the university-student sample limits generalizability, and sample composition may have influenced the relative prevalence of the profiles, particularly given the inclusion of physical education students. However, the main pattern of total physical activity differences remained after these students were excluded. Finally, the profiles were derived from only two correlated PES dimensions and were largely ordered along a low approach–high avoidance to high approach–low avoidance gradient. Because information criteria continued to improve in higher-profile solutions, the three-profile solution should be regarded as an empirical description of the present sample rather than evidence of qualitatively distinct natural subgroups. In addition, although measurement invariance was examined across gender and academic-year groups, the present study did not test measurement invariance across cultural or linguistic groups.

Future research should examine the reproducibility and longitudinal stability of the profiles in independent and more diverse samples. Longitudinal designs, latent transition analyses, and ecological momentary assessment may help determine whether physical-effort approach and avoidance tendencies and their configurations remain stable or change over time (Dunton, 2017). Further research should also directly examine cross-cultural measurement invariance (Vandenberg and Lance, 2000) and complement self-report measures of physical activity with objective indicators such as accelerometers or wearable sensors (Migueles et al., 2017). Longitudinal and experimental studies could additionally examine whether affective responses, effort-related appraisals, emotion-regulation processes, and different forms of motivational regulation prospectively relate to physical-effort approach and avoidance tendencies and subsequent physical activity. This direction is motivated by previous work linking affective and evaluative responses during exercise with subsequent exercise intentions or participation (Ekkekakis et al., 2011; Harris and Bray, 2021; Rhodes and Kates, 2015; Weyland et al., 2022). Because these processes were not directly assessed as explanatory mechanisms in the present study, they should be regarded as hypotheses for future investigation rather than mechanisms established by the current findings.

## Data Availability

The raw data supporting the conclusions of this article will be made available by the authors, without undue reservation.
