# Supplementary material for: Psychometric validation of the Chinese Physical Effort Scale and profiles of physical-effort approach and avoidance among Chinese university students
Source: Front Psychol. 2026 Oct 16;17:1959219. doi: 10.3389/fpsyg.2026.1959219 (PMC13732773; doi:10.3389/fpsyg.2026.1959219)
Supplement: Supplementary file 1 [file Supplementary_file_1.docx]

Supplementary Materials

**Chinese Version of the Physical Effort Scale (PES)** Adapted from Cheval et al. (2024).

****Instructions****

Below are a series of statements about your attitudes toward physical effort. Please indicate the extent to which you agree with each statement based on your usual feelings.

****Response options****

1 = Strongly disagree
2 = Disagree
3 = Neither agree nor disagree
4 = Agree
5 = Strongly agree

**Supplementary Table S1. Chinese version of the Physical Effort Scale (PES)**

| Dimension | Item | Chinese version |
| --- | --- | --- |
| **Approach** | PES1 | 我通常喜欢需要体力活动的项目。 |
|  | PES2 | 付出体力劳动的想法通常令我着迷。 |
|  | PES3 | 我通常享受需要体力活动的项目。 |
|  | PES4 | 我通常愿意参与需要体力活动的项目。 |
| **Avoidance** | PES5 | 我倾向于避免需要体力消耗的情况。 |
|  | PES6 | 我通常会避开需要体力消耗的任务。 |
|  | PES7 | 体力活动对我没有吸引力。 |
|  | PES8 | 我通常不喜欢需要体力活动的项目。 |

****Scoring.** Approach score:** Mean of PES1–PES4;**Avoidance score:** Mean of PES5–PES8;Higher **Approach** scores indicate a stronger tendency to approach physical effort;Higher **Avoidance** scores indicate a stronger tendency to avoid physical effort.

The original Physical Effort Scale (PES) was developed by Cheval et al. (2024). The Chinese version presented here was translated and culturally adapted following the procedures recommended by Beaton et al. (2000).

**Supplementary Figure S1.Translation and cross-cultural adaptation process**

**Supplementary Table S2. Reliability of the study measures in the pilot sample (*N* = 157)**

| Measure / subscale | No. of items | Cronbach’s α |
| --- | --- | --- |
| PES, Dimension 1 (PES-1–PES-4) | 4 | .945 |
| PES, Dimension 2 (PES-5–PES-8) | 4 | .923 |
| BREQ-3, Dimension 1 (Items 10, 11, 12, 23) | 4 | .766 |
| BREQ-3, Dimension 2 (Items 5, 6, 7, 19) | 4 | .845 |
| BREQ-3, Dimension 3 (Items 13, 20, 21, 22) | 4 | .806 |
| BREQ-3, Dimension 4 (Items 1, 2, 3, 4, 8, 9, 14, 15, 16, 17, 18) | 11 | .899 |
| ERQ, Dimension 1 (ERQ-1–ERQ-6) | 6 | .935 |
| ERQ, Dimension 2 (ERQ-7–ERQ-10) | 4 | .919 |

EFA = exploratory factor analysis; PAF = principal axis factoring; KMO = Kaiser–Meyer–Olkin measure of sampling adequacy; PES =Physical Effort Scale; BREQ-3 = Behavioral Regulation in Exercise Questionnaire-3; ERQ = Emotion Regulation Questionnaire.Reliability coefficients were calculated using the original/theoretically specified subscale composition of each measure. BREQ-3 reliability coefficients were therefore not recalculated according to the empirical factor grouping obtained in the pilot EFA.

**Supplementary Table S3. Exploratory factor analysis of the study measures in the pilot sample (*N* = 157)**

| Measure | KMO | Bartlett’s χ²(df) | p | Extraction | Rotation | No. of factors | Variance explained after PAF extraction |
| --- | --- | --- | --- | --- | --- | --- | --- |
| PES | .826 | 1161.497 (28) | <.001 | PAF | Promax | 2 | 78.47% |
| BREQ-3 | .864 | 2366.938 (253) | <.001 | PAF | Promax | 4^a^ | 58.36% |
| ERQ | .884 | 1313.766 (45) | <.001 | PAF | Promax | 2 | 73.10% |

EFA was performed using principal axis factoring with Promax rotation and Kaiser normalization. Bartlett’s tests of sphericity were significant for all three measures (p < .001). Corrected item–total correlations were calculated within the corresponding subscales. Percentages of variance explained refer to the cumulative percentages from the Extraction Sums of Squared Loadings, rather than the initial eigenvalues.

ᵃ For the BREQ-3, four factors were specified in accordance with the four-dimensional structure of the validated Chinese revised version used in the present study.

**Supplementary Table S4. Pattern coefficients from exploratory factor analyses in the pilot sample (*N* = 157)**

**Panel A. PES**

| Item | Factor 1 | Factor 2 |
| --- | --- | --- |
| PES-1 | .961 | — |
| PES-2 | .894 | — |
| PES-3 | .893 | — |
| PES-4 | .844 | — |
| PES-5 | — | .936 |
| PES-6 | — | .917 |
| PES-7 | — | .823 |
| PES-8 | — | .767 |

Factor correlation: *r* =- .482.

**Panel B. BREQ-3**

| Item | Factor 1 | Factor 2 | Factor 3 | Factor 4 |
| --- | --- | --- | --- | --- |
| BREQ-3-17 | ****.881**** | .162 | −.053 | −.140 |
| BREQ-3-3 | ****.847**** | −.059 | .158 | .013 |
| BREQ-3-1 | ****.841**** | −.167 | .140 | −.002 |
| BREQ-3-2 | ****.807**** | −.098 | .029 | .032 |
| BREQ-3-4 | ****.728**** | −.092 | .040 | .141 |
| BREQ-3-15 | ****.728**** | .039 | −.176 | .012 |
| BREQ-3-18 | ****.633**** | .168 | −.126 | .108 |
| BREQ-3-14 | ****.551**** | .119 | .237 | −.132 |
| BREQ-3-16 | ****.521**** | .038 | −.188 | .266 |
| BREQ-3-21 | .007 | ****.918**** | −.117 | .120 |
| BREQ-3-20 | .168 | ****.875**** | −.150 | −.151 |
| BREQ-3-22 | −.242 | ****.860**** | −.077 | .085 |
| BREQ-3-7 | .129 | ****.524**** | .268 | .173 |
| BREQ-3-19 | .010 | ****.476**** | .299 | −.265 |
| BREQ-3-23 | −.039 | ****.408**** | .265 | −.008 |
| BREQ-3-11 | −.128 | −.048 | ****.788**** | .005 |
| BREQ-3-12 | −.279 | −.016 | ****.710**** | .113 |
| BREQ-3-10 | .222 | −.218 | ****.698**** | .020 |
| BREQ-3-5 | −.115 | .157 | ****.594**** | .008 |
| BREQ-3-13 | .369 | .197 | ****.551**** | −.084 |
| BREQ-3-6 | −.146 | .252 | ****.511**** | .091 |
| BREQ-3-9 | .061 | −.035 | .014 | ****.911**** |
| BREQ-3-8 | .183 | .203 | .191 | ****.430**** |

Factor correlations: Factor 1–Factor 2 = −.211; Factor 1–Factor 3 = −.282; Factor 1–Factor 4 = .396; Factor 2–Factor 3 = .643; Factor 2–Factor 4 = .079; Factor 3–Factor 4 = −.026.

Note. Four factors were specified in accordance with the four-dimensional structure of the validated Chinese revised BREQ-3 used in the study. The four-factor PAF solution converged after 28 extraction iterations; Promax rotation converged after six iterations. Bold values indicate the largest absolute pattern coefficient for each item.

Panel C. ERQ

| Item | Factor 1 | Factor 2 |
| --- | --- | --- |
| ERQ-1 | .920 | — |
| ERQ-2 | .918 | — |
| ERQ-3 | .894 | — |
| ERQ-4 | .799 | — |
| ERQ-5 | .739 | — |
| ERQ-6 | .730 | — |
| ERQ-7 | — | .912 |
| ERQ-8 | — | .862 |
| ERQ-9 | — | .844 |
| ERQ-10 | — | .831 |

Factor correlation: *r* = .474.

Extraction method = principal axis factoring; rotation method = Promax with Kaiser normalization. Only salient pattern coefficients are displayed for the PES and ERQ, consistent with the SPSS output supplied for the pilot analyses.

**Supplementary Table S5. Pattern matrix for the four-factor solution of the BREQ-3 in the pilot sample**

| Item | Original dimension | Empirical factor | Primary loading |
| --- | --- | --- | --- |
| BREQ-3-17 | 4 | F1 | .881 |
| BREQ-3-3 | 4 | F1 | .847 |
| BREQ-3-1 | 4 | F1 | .841 |
| BREQ-3-2 | 4 | F1 | .807 |
| BREQ-3-4 | 4 | F1 | .728 |
| BREQ-3-15 | 4 | F1 | .728 |
| BREQ-3-18 | 4 | F1 | .633 |
| BREQ-3-14 | 4 | F1 | .551 |
| BREQ-3-16 | 4 | F1 | .521 |
| BREQ-3-21 | 3 | F2 | .918 |
| BREQ-3-20 | 3 | F2 | .875 |
| BREQ-3-22 | 3 | F2 | .860 |
| BREQ-3-7 | 2 | F2 | .524 |
| BREQ-3-19 | 2 | F2 | .476 |
| BREQ-3-23 | 1 | F2 | -.408 |
| BREQ-3-11 | 1 | F3 | -.788 |
| BREQ-3-12 | 1 | F3 | -.710 |
| BREQ-3-10 | 1 | F3 | -.698 |
| BREQ-3-5 | 2 | F3 | .594 |
| BREQ-3-13 | 3 | F3 | .551 |
| BREQ-3-6 | 2 | F3 | .511 |
| BREQ-3-9 | 4 | F4 | .911 |
| BREQ-3-8 | 4 | F4 | .430 |

**LPA Supplementary Methods**

**Model selection：**Latent profile analyses were conducted using Mplus 8.3. Models with one to six latent profiles were estimated. The optimal number of profiles was determined by considering a combination of statistical fit indices, including the Akaike Information Criterion (AIC), Bayesian Information Criterion (BIC), sample-size adjusted Bayesian Information Criterion (aBIC), entropy, the Lo–Mendell–Rubin adjusted likelihood ratio test (LMR-LRT), and the bootstrap likelihood ratio test (BLRT). In addition to statistical criteria, class size, profile interpretability, and theoretical plausibility were considered when selecting the final model.

**Classification diagnostics：**Classification quality was evaluated using entropy, average posterior probabilities for the most likely class membership, and comparison between estimated class proportions and proportions based on most likely class assignment. Average posterior probabilities greater than 0.80 were considered indicative of satisfactory classification accuracy.

**Random-start sensitivity analysis：**To evaluate whether the selected three-profile solution was sensitive to the number of random starting values, the model was estimated using three progressively larger sets of random starts (STARTS = 500 100, 1000 200, and 5000 1000). All three analyses converged normally and reproduced the same best log-likelihood (−3359.296), AIC (6738.591), BIC (6790.082), aBIC (6758.318), and entropy (.770). The best log-likelihood was replicated under all three specifications and was obtained in 2, 7, and 71 final-stage optimizations, respectively. After accounting for arbitrary class-label switching, the three analyses also yielded identical profile-specific means, profile sizes, and average posterior probabilities. Specifically, the Balanced, Avoidance-Oriented, and Approach-Oriented profiles comprised 59.07%, 14.93%, and 26.00% of the most-likely class assignments, with corresponding average posterior probabilities of .888, .900, and .907. These findings indicate that the primary three-profile solution was numerically stable with respect to the number of random starting values.

**Sensitivity to alternative variance–covariance specifications：**Greater sensitivity was observed when the within-profile variance–covariance assumptions were modified. When the variances of PES-Approach and PES-Avoidance were allowed to vary across profiles while their within-profile covariance remained fixed at zero, the model failed to terminate normally despite 5,000 EM iterations. In addition, the PES-Approach variance in one profile approached zero (estimated variance = 0.000), indicating a boundary solution. Because Mplus explicitly indicated that the resulting parameter estimates could not be trusted, fit indices, entropy, and profile-specific estimates from this model were not substantively interpreted.

In contrast, the model that retained equal indicator variances across profiles but allowed a common within-profile covariance between PES-Approach and PES-Avoidance converged normally, and the best log-likelihood was successfully replicated. This specification produced a lower AIC (6681.603), BIC (6738.244), and aBIC (6703.302) than the zero-covariance primary model. The estimated within-profile covariance between PES-Approach and PES-Avoidance was −0.608 (SE = 0.031, *p* < .001). However, the resulting profile solution differed substantially from the primary solution: approximately 98% of the sample was assigned to one dominant profile, whereas the other two profiles contained only 4 (0.31%) and 15 (1.18%) participants based on most-likely profile assignment. The profile means also no longer reproduced the Balanced, Avoidance-Oriented, and Approach-Oriented configuration identified in the primary analysis.

Taken together, these analyses indicate that the primary three-profile solution was numerically stable to substantially increased random starts but was sensitive to assumptions concerning the within-profile variance–covariance structure. In particular, allowing a within-profile association between PES-Approach and PES-Avoidance substantially altered the profile configuration, whereas allowing class-specific variances resulted in an inadmissible/nonconverged solution. Accordingly, the primary equal-variance, zero-covariance three-profile model was retained as a parsimonious and substantively interpretable descriptive representation of the joint distribution of PES-Approach and PES-Avoidance. The three profiles should therefore not be interpreted as demonstrating the existence of invariant or qualitatively discrete natural subgroups.

**Supplementary Table S6. Classification diagnostics for the selected three-profile solution**

| Profile | Estimated (%) | Assigned (%) | Average posterior probability |
| --- | --- | --- | --- |
| Balanced | 56.39 | 59.07 | .888 |
| Avoidance-Oriented | 16.32 | 14.93 | .900 |
| Approach-Oriented | 27.29 | 26.00 | .907 |

Entropy=0.770

**Supplementary Table S7.Average posterior probabilities for the selected three-profile solution**

| Most likely class | Balanced | Avoidance | Approach |
| --- | --- | --- | --- |
| Balanced | .888 | .049 | .063 |
| Avoidance | .100 | .900 | .000 |
| Approach | .093 | .000 | .907 |

Rows represent the most likely assigned latent profile, and columns represent the estimated latent profile. Diagonal values indicate the average posterior probability of correct classification.

**Supplementary Table S8. Sensitivity analysis of the selected three-profile solution**

| Statistic | STARTS = 500 100 | STARTS = 1000 200 | STARTS = 5000 1000 |
| --- | --- | --- | --- |
| Log-likelihood | −3359.296 | −3359.296 | −3359.296 |
| Best LL replicated | Yes | Yes | Yes |
| Best LL occurrences in final-stage optimizations | 2/100 | 7/200 | 71/1000 |
| AIC | 6738.591 | 6738.591 | 6738.591 |
| BIC | 6790.082 | 6790.082 | 6790.082 |
| Adjusted BIC | 6758.318 | 6758.318 | 6758.318 |
| Entropy | .770 | .770 | .770 |
| Balanced, assigned % | 59.07 | 59.07 | 59.07 |
| Avoidance-Oriented, assigned % | 14.93 | 14.93 | 14.93 |
| Approach-Oriented, assigned % | 26.00 | 26.00 | 26.00 |
| Balanced, posterior probability | .888 | .888 | .888 |
| Avoidance-Oriented, posterior probability | .900 | .900 | .900 |
| Approach-Oriented, posterior probability | .907 | .907 | .907 |

AIC = Akaike information criterion; BIC = Bayesian information criterion; aBIC = sample-size adjusted Bayesian information criterion. The increased-random-start model reproduced the same maximum log-likelihood, information criteria, entropy, profile-specific means, and most-likely profile assignments as the primary analysis. Thus, the selected solution was numerically stable with respect to the number of random starting values.

**Supplementary Table S9. Sensitivity of the three-profile solution to alternative variance–covariance specifications**

| Model | Varies | Within-profile Cov. | Cov. | LL | AIC | BIC | aBIC | **Ent.** | Smallest N (%) | Diagnosis |
| --- | --- | --- | --- | --- | --- | --- | --- | --- | --- | --- |
| M1: Primary model | Equal | Fixed at 0 | Yes | −3359.296 | 6738.591 | 6790.082 | 6758.318 | .770 | 14.93 | Three interpretable profiles |
| M2: Class-specific variances | Free | Fixed at 0 | **No** | — | — | — | — | — | — | Boundary variance = 0.000 |
| M1b: Common covariance | Equal | Equal and freely estimated | Yes | −3329.802 | 6681.603 | 6738.244 | 6703.302 | .987 | **0.31** | One dominant profile; two very small profiles |

Varies = Variances across profiles;Within-profile Cov. = within-profile covariance;Cov. = Within-profile covariance;LL = log-likelihood; AIC = Akaike information criterion; BIC = Bayesian information criterion; aBIC = adjusted Bayesian information criterion;Ent. = Entropy;Smallest *N* (%) = Smallest assigned profile (number and percentage);Diagnosis = Main diagnostic indicator.

**Supplementary Table S10. Profile characteristics under the primary and common-covariance specifications**

| Model / Profile | PES-Approach mean | PES-Avoidance mean | Assigned n | Assigned % |
| --- | --- | --- | --- | --- |
| **M1: Primary specification** |  |  |  |  |
| Avoidance-Oriented | 1.753 | 4.028 | 190 | 14.93 |
| Balanced | 3.107 | 3.140 | 752 | 59.07 |
| Approach-Oriented | 4.452 | 2.209 | 331 | 26.00 |
| **M1b: Common-covariance specification** |  |  |  |  |
| Profile 1 | 3.239 | 3.013 | 1254 | 98.51 |
| Profile 2 | 1.281 | 1.457 | 4 | 0.31 |
| Profile 3 | 4.886 | 4.738 | 15 | 1.18 |

Profile labels from the primary model were assigned according to the relative levels of PES-Approach and PES-Avoidance. Profiles from the common-covariance sensitivity model were intentionally left unlabeled because they did not reproduce the substantive Balanced, Avoidance-Oriented, and Approach-Oriented configuration observed in the primary model. In M1b, the within-profile covariance between PES-Approach and PES-Avoidance was −0.608 (SE = 0.031, p < .001), with equal within-profile variances of 0.966 for PES-Approach and 0.941 for PES-Avoidance.

**Supplementary Table S11. Sensitivity Analyses After Excluding Physical Education Students**

Panel A. Three-profile solution after excluding physical education students

| Profile | *n* (%) | Physical-effort approach, *M* (*SE*) | Physical-effort avoidance, *M* (*SE*) |
| --- | --- | --- | --- |
| Balanced | 640 (61.07) | 3.066 (0.046) | 3.178 (0.036) |
| Avoidance-Oriented | 174 (16.60) | 1.723 (0.064) | 4.106 (0.076) |
| Approach-Oriented | 234 (22.33) | 4.402 (0.046) | 2.160 (0.110) |

*N* = 1,048 after excluding 225 physical education students. Entropy = .780. Profile labels were assigned according to the relative levels of physical-effort approach and avoidance rather than the numerical class labels generated by Mplus.

Panel B. BCH comparisons of physical activity outcomes after excluding physical education students

| Outcome | BAL *M* (SE) | AVOM (SE) | APP *M* (SE) | Overall BCH χ²(2) | *p* | APP vs. BAL | APP vs. AVO | BAL vs. AVO |
| --- | --- | --- | --- | --- | --- | --- | --- | --- |
| Total PA | 1958.90 (55.66) | 1987.76 (111.57) | 2619.85 (124.34) | 22.080 | < .001 | < .001 | < .001 | .828 |
| MVPA | 1000.62 (41.46) | 973.18 (81.75) | 1551.80 (100.36) | 25.620 | < .001 | < .001 | < .001 | .778 |

BAL = Balanced; AVO = Avoidance-Oriented; APP = Approach-Oriented; PA = physical activity; MVPA = moderate-to-vigorous physical activity; BCH = Bolck–Croon–Hagenaars procedure. Physical activity outcomes are expressed as MET-min/week. Values are BCH-adjusted estimated means, with standard errors in parentheses.

**Supplementary Appendix S1. Mplus syntax used for latent profile analysis**

TITLE:

PES LPA 3-Class Model;

DATA:

FILE IS LPAdata.dat;

VARIABLE:

NAMES ARE

gender

grade

PES_Approach

PES_Avoidance

ERQ_Reappraisal

ERQ_Suppression

BREQ_AM

BREQ_ER

BREQ_IR

BREQ_AUT

Total_MET;

USEVARIABLES ARE

PES_Approach

PES_Avoidance;

CLASSES = c(3);

ANALYSIS:

TYPE = MIXTURE;

ESTIMATOR = MLR;

STARTS = 500 100;

STITERATIONS = 20;

LRTSTARTS = 0 0 500 100;

OUTPUT:

TECH1 TECH4 TECH11 TECH14;

**Supplementary Appendix S2. Mplus syntax for the increased-random-start primary model**

TITLE:

PES LPA 3-Class Model - Increased Random Starts;

DATA:

FILE IS LPAdata.dat;

VARIABLE:

NAMES ARE

gender

grade

PES_Approach

PES_Avoidance

ERQ_Reappraisal

ERQ_Suppression

BREQ_AM

BREQ_ER

BREQ_IR

BREQ_AUT

Total_MET;

USEVARIABLES ARE

PES_Approach

PES_Avoidance;

CLASSES = c(3);

ANALYSIS:

TYPE = MIXTURE;

ESTIMATOR = MLR;

STARTS = 5000 1000;

STITERATIONS = 20;

OUTPUT:

TECH1 TECH4;

**Supplementary Appendix S3. Mplus syntax for the class-specific variance model**

TITLE:

PES LPA 3-Class Model - Class-Specific Variances;

DATA:

FILE IS LPAdata.dat;

VARIABLE:

NAMES ARE

gender

grade

PES_Approach

PES_Avoidance

ERQ_Reappraisal

ERQ_Suppression

BREQ_AM

BREQ_ER

BREQ_IR

BREQ_AUT

Total_MET;

USEVARIABLES ARE

PES_Approach

PES_Avoidance;

CLASSES = c(3);

ANALYSIS:

TYPE = MIXTURE;

ESTIMATOR = MLR;

STARTS = 5000 1000;

STITERATIONS = 20;

MITERATIONS = 5000;

MODEL:

%OVERALL%

%c#1%

PES_Approach;

PES_Avoidance;

%c#2%

PES_Approach;

PES_Avoidance;

%c#3%

PES_Approach;

PES_Avoidance;

OUTPUT:

TECH1 TECH4;

**Supplementary Appendix S4. Mplus syntax for the common within-profile covariance model**

TITLE:

PES LPA 3-Class Model - Equal Variances with Common Covariance;

DATA:

FILE IS LPAdata.dat;

VARIABLE:

NAMES ARE

gender

grade

PES_Approach

PES_Avoidance

ERQ_Reappraisal

ERQ_Suppression

BREQ_AM

BREQ_ER

BREQ_IR

BREQ_AUT

Total_MET;

USEVARIABLES ARE

PES_Approach

PES_Avoidance;

CLASSES = c(3);

ANALYSIS:

TYPE = MIXTURE;

ESTIMATOR = MLR;

STARTS = 5000 1000;

STITERATIONS = 20;

MITERATIONS = 5000;

MODEL:

%OVERALL%

PES_Approach WITH PES_Avoidance;

OUTPUT:

TECH1 TECH4;
